# Emerging Treatment Options for Sarcopenia in Chronic Liver Disease

**DOI:** 10.3390/life11030250

**Published:** 2021-03-17

**Authors:** Yun Kim

**Affiliations:** Hanyang Medicine-Engineering-Bio Collaborative & Comprehensive Center for Drug Development, Hanyang University, Seoul 04763, Korea; mn0701@hanyang.ac.kr; Tel.: +82-2-2290-9664 or +82-10-2013-9226

**Keywords:** sarcopenia, chronic liver disease, muscle protein turnover, cirrhosis, non-alcoholic fatty liver disease

## Abstract

Sarcopenia is characterized by a skeletal muscle disorder with progressive and generalized loss of muscle mass and function, and it increases the risk of adverse outcomes with considerable prevalence in patients with chronic liver disease. Sarcopenia in chronic liver disease underlies complicated and multifactorial mechanisms for pathogenesis, including alterations in protein turnover, hyperammonemia, energy disposal, hormonal changes, and chronic inflammation. The key contribution to sarcopenia in patients with chronic liver diseases can be the hyperammonemia-induced upregulation of myostatin, which causes muscle atrophy via the expression of atrophy-related genes. Several clinical studies on emerging treatment options for sarcopenia have been reported, but only a few have focused on patients with chronic liver diseases, with mostly nutritional and behavioral interventions being carried out. The inhibition of the myostatin-activin receptor signaling pathway and hormonal therapy might be the most promising therapeutic options in combination with an ammonia-lowering approach in sarcopenic patients with chronic liver diseases. This review focuses on current and emerging treatment options for sarcopenia in chronic liver diseases with underlying mechanisms to counteract this condition.

## 1. Introduction

Sarcopenia is characterized by a skeletal muscle disorder with progressive and generalized loss of muscle mass, strength, and function, thus increasing the risk of adverse outcomes, such as physical disability and higher rates of hospitalization and mortality [1]. The term “sarcopenia” was first used in the 1980s to refer to age-related skeletal muscle decline [2]. The definition of sarcopenia has changed from muscle-wasting conditions with low muscle mass into a term involving muscle function in the current concept of sarcopenia [3,4]. The evolution of the definition is attributed to the fact that muscle function has been shown to be a more influential clinical biomarker than muscle mass alone [5,6]. The overall prevalence of sarcopenia was estimated at 10% in both men and women, which indicates that a considerable proportion of the elderly, even in a healthy population, has sarcopenia [7]. Moreover, sarcopenia is generally known to be associated with various chronic inflammatory states, including chronic liver disease [6]. It has been shown to be a significant risk factor for non-alcoholic fatty liver disease (NAFLD), regardless of obesity or metabolic syndrome [8,9,10]. This relationship was convincing because similar pathological factors, including insulin resistance and inflammation, exist between sarcopenia and NAFLD [11,12,13]. Notably, the prevalence rate of sarcopenia is assumed to be 30–70% in cirrhotic patients, with a higher rate among men than in women (61.6% vs. 36%, respectively) [14,15,16]. Minimal hepatic encephalopathy, a complication of liver cirrhosis, was significantly associated with the presence of either muscle mass loss or strength loss (60.9% vs. 37.7%, respectively) [17]. In addition, sarcopenia commonly develops in patients with end-stage liver disease, for which the prevalence of sarcopenia ranges from 14 to 78% and from 30 to 100% in patients before and after liver transplantation, respectively [18]. Furthermore, sarcopenia can be a clinically significant predictor of higher rates of mortality and infection [16,19], longer hospitalization [20], and increased economic burden [21], thus reducing the quality of life [22]. However, challenges arise because the mechanisms of sarcopenia in chronic liver diseases are poorly understood and no approved and effective therapeutics to counteract sarcopenia are available. Therefore, in this review, I focused on the current and emerging treatment options for sarcopenia in chronic liver diseases with underlying mechanisms to counteract this condition.

## 2. Etiology of Sarcopenia in Chronic Liver Diseases

**Figure 1:**
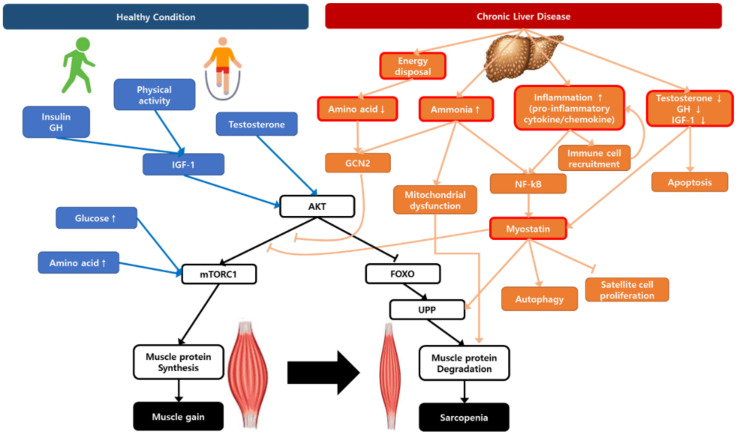
A schematic representation illustrating the regulation of muscle protein synthesis and degradation with the contribution of chronic liver disease to sarcopenia. AKT, protein kinase B; FOXO, Forkhead box O; GCN2, general control non-depressed 2; GH, growth hormone; IGF-1, insulin growth factor-1; mTORC1, mammalian target of rapamycin complex 1; NF-κB, nuclear factor-κB; UPP, ubiquitin–proteasome pathway.

Sarcopenia in chronic liver disease is a complicated and multifactorial disease with several main drivers, such as impairment in protein turnover, malnutrition, hyperammonemia, chronic inflammation, and hormonal changes (Figure 1). Understanding the hypotheses of sarcopenia development would play a key role in overcoming the therapeutic limitations.

### 2.1. Alterations in the Protein Turnover

The balance between protein synthesis and degradation in skeletal muscles ensures the maintenance of protein turnover [23]. Skeletal muscle protein turnover can be regulated by several factors, including food intake, fasting, endocrine changes (i.e., insulin levels and resistance, insulin-like growth factor 1 (IGF-1), testosterone, and corticosteroids), myostatin, cytokines, and physical activity [24,25]. Some key molecular pathways that explain muscle protein turnover include the following: Akt-mediated mammalian target of rapamycin complex 1 (mTORC1) signaling, satellite cell signaling, and ubiquitin–proteasome signaling pathways [26,27]. Muscle mass is positively regulated via mTORC1 with several factors, such as growth factors, insulin or IGF-1, and energy status (glucose and amino acids) [28]. In addition, satellite cell signaling pathways contribute to muscle regeneration and growth via myonuclear accretion [29]. Interleukin 6 (IL-6) may induce satellite cell proliferation and the IGF-1 that is required for a growth stimulus activates the IGF-1–Akt pathway in satellite cells, both of which results in muscle hypertrophy [30,31]. Myostatin, which is a member of the transforming growth factor β (TGFβ) superfamily, negatively regulates the satellite cell activation and self-renewal that contributes to muscle protein degradation via inhibition of the Akt pathway for protein synthesis and an increase in the ubiquitin–proteasome system to cause muscle atrophy [32,33]. Myostatin has been observed as a regulator of the catabolic pathway in skeletal muscle via the ubiquitin–proteasome and autophagy–lysosome pathways. Additionally, it has been shown to downregulate the Akt/forkhead box O (FOXO; transcription factor) pathway, causing muscle atrophy through the expression of atrophy-related genes (atrogenes) [33].

In patients with chronic liver diseases, controversial results from whole-body turnover studies have been published. As expected, some previous studies indicated that muscle wasting in cirrhosis could occur due to the decrease in muscle protein synthesis using different methods of arteriovenous exchanges and a whole-body trace [34,35,36]. However, other whole-body turnover studies with phenylalanine and leucine labeling reported that muscle protein turnover increased or remained unchanged [37,38]. These contradictory observations may be attributed to the lack of homogeneity in the methodology and clinical characteristics, including the etiology, age, and severity of liver diseases [15].

### 2.2. Hyperammonemia

Hyperammonemia, which is the increase in blood ammonia, in advanced liver diseases is a result of liver dysfunction accompanied by blood shunted around the liver and impaired ureagenesis [39,40]. Since skeletal muscle acts as a primary site for the depletion of extrahepatic ammonia, muscle wasting commonly occurs in patients with chronic liver diseases [39,41,42].

The mechanisms of muscle mass depletion that are related to hyperammonemia can be explained as follows. Ammonia levels in skeletal muscle are substantially elevated in patients with cirrhosis, thus resulting in the induction of the transcription factor NF-κB and a further increase in the myostatin expression, followed by the inhibition of myogenesis and an increase in autophagy [41,42]. Therefore, the key contribution to sarcopenia in patients with chronic liver disease is the hyperammonemia-induced upregulation of myostatin. In addition, the removal of ammonia occurs in skeletal muscles through the synthesis of glutamine, which is exchanged for branched-chain amino acids (BCAAs), such as leucine [43]. This also explains the decreased tendency of plasma BCAAs in patients with cirrhosis [44]. Mitochondrial dysfunction can also explain the contribution of hyperammonemia to the decrease in muscle protein synthesis. Because of higher ammonia levels in the muscle, cataplerosis (the removal of intermediate metabolites in the tricarboxylic acid cycle) dominantly occurs and induces a decrease in alpha-ketoglutarate, resulting in mitochondrial dysfunction [15,41]. Furthermore, hyperammonemia has been reported to induce oxidative stress by elevating the amount of reactive oxygen species (ROS) [45]. In conditions with high levels of ammonia, the generation of ROS can lead to increased amounts of carbonylated proteins and thiobarbituric acid-reactive substances in skeletal muscle [45]. Given the high prevalence of cirrhosis, the abovementioned studies suggest that ammonia-lowering approaches, NF-κB inhibitors, myostatin inhibitors, and anaplerotic substrates against mitochondrial dysfunction are potential therapeutics to counteract sarcopenia in chronic liver diseases.

### 2.3. Energy Disposal

Patients with advanced liver diseases, such as cirrhosis, are in an accelerated state of starvation, with increased gluconeogenesis, fat oxidation, ketogenesis, and a catabolic state [46,47,48,49]. In conditions of increased gluconeogenesis, circulating BCAAs in skeletal muscle are likely to decrease due to increased utilization as an energy source [49]. Reduced amino acid levels promote adaptive cellular homeostasis with elevated autophagy in skeletal muscles. Additionally, intracellular amino acid deprivation induces an integrated stress response that is mediated via activation of the amino acid deficiency sensor known as general control non-depressed 2 (GCN2) [50,51]. The activated sensor further phosphorylates the eukaryotic initiation factor 2α (eIF2α), resulting in the upregulation of the activating transcription factor 4 (ATF4), which induces the expression of Sestrin2 (stress response protein) and the sustained inhibition of mTORC1 to preserve amino acid levels [50,52]. Thus, the combined contribution of GCN2 and mTORC1 regulates the amino acid homeostasis and subsequently restores the global protein synthesis [50,53].

Patients with cirrhosis and hyperammonemia experience a similar condition of integrated stress response due to amino acid deprivation. However, hyperammonemia persistently activates GCN2 with an impaired translation of ATF4, which results in decreased muscle protein synthesis and impaired proteostasis [54,55]. Thus, patients with hyperammonemia may detour to alternative pathways of an adaptive integrated stress response, which is supposed to be mediated by the leucine exchanger SLC7A5/LAT1 [55,56]. The expression of SLC7A5/LAT1 was increased in patients with cirrhosis and it seems to play a role in increasing leucine uptake [55,56]. Subsequently, increased leucine concentration can be utilized in the mitochondria for energy output and can prevent the breakdown of GCN2-mediated protein synthesis as an adaptive response.

### 2.4. Hormonal Changes

Alterations in the endocrine system, such as hypotestosteronemia and an impaired insulin/IGF-1 pathway, are associated with an advanced liver disease, which could also contribute to muscle wasting [57]. Up to 90% of men with cirrhosis exhibit reduced serum testosterone, which is proportional to the decrease in liver function [58,59]. Additionally, testosterone deficiency has been shown to be an independent clinical biomarker in liver cirrhosis, and lower levels of testosterone have been identified in sarcopenic patients with cirrhosis compared to those without sarcopenia [58,60]. Hypogonadism, an abnormality in the hypothalamic–pituitary–gonadal axis, is involved in sarcopenia and chronic liver disease and contributes to low testosterone levels, further increasing mortality [61]. Since the anabolic influence of testosterone is well known and important in skeletal muscles, testosterone might reverse sarcopenia via multiple signaling pathways, including the suppression of myostatin and muscle cell apoptosis and the stimulation of proliferation pathways in muscle remodeling [62].

Growth hormone (GH) deficiency is correlated with fatty liver disease and the impairment of cell metabolism, which contributes to the further development of chronic liver disease [63]. GH primarily regulates the production of IGF-1, and the anabolic effect on skeletal muscles arises from both GH and IGF-1 [64]. Besides low serum levels of testosterone, a decreased IGF-1 level has been demonstrated as one of the potential risk factors for sarcopenia because IGF-1 contributes to the proliferation of satellite cells and increases muscle protein synthesis via the activation of the Akt/mTORC1 signaling pathway [65,66,67]. Furthermore, IGF-1 inhibits the proteolysis and activation of muscle-atrophy-related ubiquitin ligases, such as atrogin-1 (MAFbx) and MuRF-1 [68]. In elderly European men aged ≥70 years, low baseline IGF-1 was related to a greater reduction in gait speed; however, it is necessary to identify whether the replacement of IGF-1 is effective and safe to reverse sarcopenia in randomized controlled studies [69].

### 2.5. Inflammation, NAFLD, and Obesity

NAFLD is a common liver disease worldwide that can subsequently progress to non-alcoholic steatohepatitis (NASH), liver fibrosis, cirrhosis, and hepatocellular carcinoma [70]. In particular, NASH is characterized by hepatic steatosis, inflammation, and liver cell damage [71]. In addition to chronic inflammation, which is currently observed to have a direct relationship with the severity of NAFLD, insulin resistance and physical inactivity are important risk factors that are related to the development of both sarcopenia and NAFLD [11,72]. Hepatic lipotoxicity and non-liver factors, such as inflammation in adipose tissues, are linked to the elevation of proinflammatory cytokines and chemokines as key markers of NASH, such as tumor necrosis factor α (TNF-α), IL-6, chemokine CC ligand-2, and C-reactive protein [72,73,74]. Following adipocyte enlargement in NAFLD, macrophage recruitment and polarization in the proinflammatory state in adipose tissues increase various proinflammatory signals, such as adipokines, contributing to the progression of NAFLD [75,76]. Among the inflammatory cytokines, TNF-α plays a key role in the alteration of muscle protein turnover in patients with liver disease [77,78,79]. TNF-α may alter the phosphorylation of the mTORC1 pathway, thus decreasing muscle protein synthesis, and might also induce the ubiquitin–proteasome system and the expression of atrogin-1, resulting in muscle protein breakdown [77,78,79,80]. Furthermore, TNF-α-induced skeletal muscle atrophy could be explained by the ceramide accumulation, which is related to sphingolipid metabolism, and by the activation of NF-κB due to ROS elevation, which increases muscle protein degradation [81,82,83]. Altogether, the elevation of circulating inflammatory cytokines may contribute to muscle wasting in liver diseases, and decreasing cytokine levels might be a pharmacological approach to attenuate the loss of skeletal muscle mass.

Obesity is correlated with increased fatty acids, which causes oxidative stress and autophagy, along with the accumulation of lipid intermediates in skeletal muscles [84,85,86]. The accumulation of lipid intermediates, including diacylglycerol and ceramides, into the skeletal muscle (termed myosteatosis) can result in insulin resistance and mitochondrial dysfunction via impaired β-oxidation capacity [84,85,86]. Myosteatosis can lead to muscle atrophy, and the extent of lipid infiltration is negatively correlated with muscle function and regeneration [87,88]. Sarcopenic obesity is characterized by the accumulation of excessive adipose tissues, along with a decline in lean body mass [89]. Similar to myosteatosis, obese adipocytes induce the accumulation of proinflammatory immune cells, including macrophages and lymphocytes, as well as the abnormal production of diverse adipokines, which leads to the development of local proinflammatory conditions [89]. Leptin, one of the secreted adipokines from inflamed adipose tissue, contributes to the loss of skeletal muscle [90]. Furthermore, inflammatory cytokines of TNF-α and IL-6 induce insulin resistance via inhibition of the insulin receptor activity and signaling, along with decreased glucose uptake [91]. An increased level of leptin stimulates the production of TNF-α and IL-6, resulting in a vicious cycle [89]. Other obesity-induced inflammatory factors include resistin and retinol-binding protein 4, which inhibits insulin signaling and contributes to insulin resistance, respectively [92,93,94]. In addition, obesity can induce a decreased level of fibroblast growth factor 21 and adiponectin, which results in decreased insulin signaling and β-oxidation in the liver [95,96]. Therefore, obesity could be a crucial factor for the complex mechanisms of pathophysiology between sarcopenia and NAFLD [97].

Altogether, intramuscular lipids have detrimental effects on muscle function, including mitochondrial dysfunction, oxidative stress that contributes to lipotoxic conditions, and insulin resistance, along with the release of proinflammatory myokines. Therefore, in a vicious cycle of inflammation, the exacerbated inflammation in adipose tissues and skeletal muscles triggers the development of sarcopenic obesity and NAFLD. To the best of our knowledge, the effects of lipid derivatives in patients with chronic liver disease and sarcopenia have not yet been studied.

## 3. Current and Emerging Treatment Options for Sarcopenia in Chronic Liver Disease

In recent decades, several drugs have been investigated in clinical trials to counteract sarcopenia, but no pharmacologically effective therapeutics have been approved to date. However, current and emerging treatment options for sarcopenia have been reported to be under development. In February 2021, a manual search from the Clinicaltrials.gov database (accessed on 1 February 2021) yielded several published interventional clinical trials related to sarcopenia and the potential expanded use with mostly late-phase studies (Table S1). In addition, the list of clinical trials studying therapeutic interventions for sarcopenia in chronic liver disease is provided in Table 1, which mostly consists of dietary supplements and/or behavioral interventions. Unfortunately, there is only one registered interventional clinical trial of a pharmacological treatment option using testosterone, indicating that the development of a new drug is urgently needed (Table 1). The following section discusses an update on the potential pharmacological treatment options for the treatment of sarcopenia with chronic liver diseases.

### 3.1. Hormonal Treatment

Since testosterone deficiency is a common feature in advanced liver diseases, a previous study reported that testosterone treatment can reduce fat mass and hemoglobin A1c and can increase muscle and bone mass, along with hemoglobin elevation in patients with cirrhosis [61]. Testosterone treatment particularly increases the expression of androgen receptors, resulting in muscle cell growth and the differentiation for muscle protein synthesis [98]. In addition, testosterone drives the upregulation of IGF-1 via the Akt pathway to enhance beneficial effects on muscle growth via the proliferation of satellite cells [62,99]. By means of another pathway, testosterone replacement therapy contributes to the myostatin downregulation, further suppressing apoptosis in skeletal muscles [62]. However, clear molecular evidence of testosterone treatment should be obtained via further research, and adverse events, such as cardiovascular diseases, fluid retention, gynecomastia, sleep apnea, and the progression of prostatic diseases, need to be cautiously monitored [100]. Therefore, long-term confirmatory studies are needed to prove its efficacy and safety in sarcopenic patients with chronic liver diseases [101].

As previously mentioned, GH deficiency is correlated with the development of chronic liver diseases, which supports the hypothesis that GH replacement treatment can improve muscle mass by increasing serum IGF-1 levels and IGF binding protein 3 with the activation of the mTORC1 signaling pathway [102,103,104]. GH replacement therapy may also involve antioxidant defenses through the activation of mitochondrial biogenesis pathways [104]. However, GH supplementation may cause a high rate of adverse reactions, including worsening ascites and edema, with limited applicability due to its high cost [104,105]. Therefore, the clinical utility of GH replacement treatment needs to be confirmed in further studies to identify its safety and efficacy in clinical use.

### 3.2. Myostatin and Activin Receptor

Myostatin is an important target in various studies because of its detrimental effects on muscle protein synthesis [106]. Myostatin inhibits the differentiation and growth of skeletal muscle cells by binding to the activin type IIB (ACVRIIB) receptor, which subsequently inhibits the differentiation of myoblasts and the mTORC1 signaling pathway [107]. Stamulumab (MYO-029), a myostatin inhibitor studied in human trials, is a recombinant human antibody that neutralizes myostatin, which inhibits its binding to ACVRIIB. However, further development was stopped due to the limited efficacy on muscle strength in phase 2 clinical trials in patients with muscular dystrophy. Landogrozumab (LY-2495655), another myostatin inhibitor as a humanized monoclonal antibody under review, also binds to myostatin and neutralizes its activity. Landogrozumab has been shown to increase total lean body mass with a fat mass reduction in older weak fallers and to improve general physical performance [108]. Clinical trials of landogrozumab treatment for muscle atrophy in patients with hip arthroplasty identified improvement in muscle mass; however, the lean body mass did not meet the threshold [109]. Trevogrumab (REGN1033) is another human monoclonal antibody that targets myostatin for the treatment of sarcopenia, where its safety and efficacy are still being assessed after the completion of phase 2 clinical trials [110].

Ramatercept (ACE-031), an ACVRIIB/Fc recombinant fusion protein, binds to the ligands (e.g., myostatin, activins, and growth differentiation factor 11) of ACVRIIB to inhibit the endogenous receptor binding. Despite its award of orphan designation and accelerated review by the U.S. Food and Drug Administration (FDA), further development for the treatment of muscular dystrophy was stopped after completion in 2011 because of safety concerns, such as minor nosebleeds, gum bleeding, and/or small dilated blood vessels within the skin [111,112]. ACE-083, as an alternative form of ACE-031, is a locally acting and follistatin-based fusion protein that binds and acts by neutralizing myostatin, activins, and growth differentiation factor 11 [113]. Follistatin is known to improve muscle growth and function by preventing ligands from binding to receptors [114]. A first-in-human phase I clinical trial of ACE-083 demonstrated that it was well tolerated and produced increased muscle volume in healthy volunteers, which provides evidence for the potential treatment of various neuromuscular disorders, along with the need for further investigation of its efficacy and safety [115].

ACVRIIB is another potentially effective target for the development of treatments for sarcopenia. Bimagrumab (BYM-338), a human monoclonal antibody targeting ACVRIIB, was designed to competitively bind to ACVRIIB with higher affinity than its ligands. Breakthrough therapy designation was granted to bimagrumab in 2013 by the FDA for sporadic inclusion body myositis, which is characterized by inflammatory myopathy and progressive skeletal muscle atrophy. A preclinical study showed that bimagrumab increased the differentiation of myoblasts and inhibited the activity of myostatin or activin A, thus resulting in the improvement of skeletal muscle mass in mice [116]. A phase 2 clinical trial of bimagrumab in elderly patients with sarcopenia and limited mobility showed that bimagrumab improved muscle growth/function and mobility [117]. However, other late phases of clinical trials for bimagrumab treatment increased skeletal muscle mass in one study but observed no significant effects on functional capacity in sarcopenic patients with chronic obstructive pulmonary disease or sporadic inclusion body myositis [118,119]. In addition, the clinical use of ACVRIIB inhibitors may cause several adverse events, including muscle spasms, diarrhea, and acne [117,118,119]. Although there are many past and ongoing studies showing that the inhibition of the myostatin/ACVRIIB signaling pathway may counteract sarcopenia, the combination approaches with nutritional and/or physical activity could be a more promising and effective treatment for sarcopenia [110,120]. While this therapeutic approach has not yet been studied in sarcopenic patients with chronic liver diseases, the current status of research indicates that myostatin/ACVRIIB signaling inhibition can be an emerging treatment option for muscular dystrophy.

### 3.3. Ammonia-Lowering Treatment

As previously discussed, hyperammonemia is a feature of patients with cirrhosis that contributes to abnormal skeletal muscle proteostasis. Although the clinical utility of ammonia-lowering treatment is expected to be effective, whether this therapeutic approach can improve proteostasis and reverse sarcopenia in chronic liver disease is uncertain. A preclinical study showed that ammonia-lowering treatment significantly increased lean body mass and improved grip strength and skeletal muscle growth [121]. Perturbed molecular actions due to hyperammonemia were also improved with the reduction of myostatin expression and autophagy markers and with the reversal of GCN2/eIF2α phosphorylation [121]. L-ornithine L-aspartate can be an adequate option for ammonia-lowering treatment for patients with cirrhosis suffering from hepatic encephalopathy through the improvement of skeletal muscle growth and function, as supported by several randomized clinical trials and meta-analyses [122]. In addition, nutraceuticals, such as BCAA, L-carnitine, omega-3 polyunsaturated fatty acids, zinc, and vitamin D, may provide a promising standard of care with beneficial improvements in muscle homeostasis for sarcopenia in chronic liver disease [123]. Confirmation of these ammonia-lowering approaches for the treatment of sarcopenia in chronic liver disease is necessary for powered and well-controlled clinical trials to provide further evidence of efficacy.

### 3.4. Clinical Nutrition

In cirrhotic patients, the rates of both hepatic glucose production and oxidation are decreased owing to a depletion of hepatic glycogen, although gluconeogenesis is increased [124,125]. Thus, patients are susceptible to an accelerated state of starvation after an overnight fast [49]. Furthermore, impaired protein turnover and decreased plasma levels of essential fatty acids are observed in cirrhosis [126,127]. Therefore, the European Society for Clinical Nutrition and Metabolism (ESPEN) guideline recommends that the starvation period be kept short in cirrhosis to ameliorate protein turnover by taking 3–5 meals/day and a late evening snack [128]. A late evening snack has been shown to improve the nitrogen balance and decreased lipid oxidation, regardless of the composition or type of formulation used [129,130]. It is also suggested that cirrhotic patients with sarcopenia should include an optimal energy intake of 30–35 kcal/kg/day and a target protein intake of 1.2–1.5 g/kg/day [128]. To overcome protein depletion in cirrhotic patients with sarcopenia, including those with sarcopenic obesity, increased protein intake can improve protein anabolism and the status of total body protein [128,131,132]. In addition, in cirrhotic patients, including those with advanced cirrhosis and a previous episode of hepatic encephalopathy, a long-term BCAA supplementation (0.20–0.25 g/kg/day) had beneficial effects on protein metabolism, resulting in improved muscle mass, as well as minimal hepatic encephalopathy [133,134,135,136]. Since a specific nutritional intervention is needed in sarcopenic patients with chronic liver disease, multidisciplinary nutrition care should be implemented in the metabolic management of patients to achieve nutritional goals.

### 3.5. Regenerative Therapeutic Approach: Mitochondrial Restoration and Anti-Inflammation

Since the current pharmaceutical options for sarcopenia in chronic liver diseases may be ineffective and restricted in terms of the available clinical evidence, novel therapeutic approaches are necessary to improve mitochondrial function, reduce chronic inflammation, and induce muscle tissue regeneration, thus leading to increased muscle growth and function. Considering the abovementioned etiology of sarcopenia, regenerative medicine and stem cell therapy are potential alternatives for sarcopenia alleviation because of their ability to change the proinflammatory microenvironment into regenerating and reinnervating conditions by producing anti-inflammatory cytokines [137]. Mesenchymal stem cell transplantation has been shown to modulate immunological effects through the production of anti-inflammatory cytokines, including IL-10 and IL-13, and to stimulate neurosupportive effects by secreting factors including basic fibroblast growth factor and vascular endothelial growth factor [138,139,140,141,142]. In addition, mesenchymal stem cells may restore mitochondrial function in skeletal muscle via the mediation of mitochondrial transplantation [143]. However, stem cell transplantation has many restrictive hurdles to overcome (e.g., controversial safety and efficacy, ethics, pharmaceutical manufacturing process, and quality control); the secretome of stem cells that houses the important anti-inflammatory agents may provide a more promising option than the direct use of stem cells [137]. Nevertheless, extensive research through preclinical and clinical studies with a larger patient population is still required to determine its efficacy and safety as a potential therapeutic option for sarcopenia in chronic liver disease.

## 4. Conclusions

Sarcopenia in chronic liver disease is a complicated and multifactorial disease with various contributing factors. Several clinical studies on sarcopenia in chronic liver diseases have examined the effects of nutritional supplements, behavioral interventions, and their combinations, but pharmacological therapeutic approaches have rarely been studied. Since the attempt of a nutritional approach is not always effective in improving clinical outcomes, behavioral intervention is practically impossible for bedridden patients who may need these approaches the most. Therefore, there is an urgent need to develop potential treatment options, including an ammonia-lowering approach that blocks the myostatin–activin receptor pathway, as well as hormonal therapy, regenerative therapeutics, and their combinations, to prevent and reverse sarcopenia.

## Figures and Tables

**Table 1:** Current and emerging therapeutic interventions for sarcopenia with chronic liver diseases observed in clinical trials.

Target or Mechanism of Action	Intervention	Sponsor/Collaborator	Clinical Phase	Indication	Status	NCT Number	Year		Title
							Start	End	
Testosterone	Testosterone undecanoate	Institute of Liver and Biliary Sciences, India	NA	Liver cirrhosis	Recruiting	NCT03995251	2019	2020	Efficacy and Safety of Testosterone Therapy in Improving Sarcopenia in Men with Cirrhosis: A Randomized Controlled Trial
Behavior	Exercise	University of California, San Francisco/Johns Hopkins University, Duke University	NA	End-stage liver disease, sarcopenia, liver cirrhosis	Completed	NCT02367092	2016	2019	Exercise Intervention in Liver Transplant Patients
Behavior	Exercise	Memorial Hospital Groups	NA	End-stage liver disease, chronic liver failure, sarcopenia	Completed	NCT04546048	2018	2019	The Early Strength Training Exercise Therapy in Liver Recipients: Protocol for an Observational Feasibility Trial
Behavior	Pulmonary rehabilitation exercise, home-based exercise	Mayo Clinic	NA	End-stage liver disease	Recruiting	NCT03266575	2018	Ongoing	Does Pulmonary Rehabilitation Improve Frailty and Sarcopenia in End-Stage Liver Disease?
Dietary supplement	Amino acid infusion	Rigshospitalet, Denmark/Hvidovre University Hospital	NA	Cirrhosis	Completed	NCT02132962	2014	2015	Sarcopenia and Cirrhosis
Dietary supplement	BCAA	Puerta de Hierro University Hospital	NA	Sarcopenia	Completed	NCT04073693	2017	2019	Characterization of the Nutritional Status in the Patient with Liver Cirrhosis and Impact of a Nutritional Intervention with Nutritional Supplements with BCAA vs. Standard Treatment in the Subgroup of Patients with Sarcopenia
Dietary supplement	BCAA	Dayanand Medical College and Hospital	4	Liver cirrhosis	Recruiting	NCT03633279	2018	2020	Treatment of Sarcopenia Improves the Muscle Mass and Muscle Strength of Patients with Liver Cirrhosis—Child C: A Randomized Double Blind Control Trial
Dietary supplement	BCAA	Institute of Liver and Biliary Sciences, India	NA	Chronic liver disease	Recruiting	NCT04246918	2020	Ongoing	Effect of Branched Chain Amino Acids Supplementation on Muscle Mass, Muscle Quality, and Molecular Markers of Muscle Regeneration in Patients With Chronic Liver Disease: A Randomized Controlled Trial
Dietary supplement	HMB	University of Roma La Sapienza	NA	Sarcopenia	Completed	NCT03234920	2015	2018	Effects of β-Hydroxy-β-methylbutyrate (HMB) Supplementation after Liver Transplantation: Randomized and Controlled Pilot Study
Dietary supplement	CaHMB	Shanghai Zhongshan Hospital	NA	Sarcopenia, liver cirrhosis	Unknown	NCT03605147	2018	2019	The Effect of Calcium β-Hydroxy-β-methylbutyrate Supplementation in Sarcopenia in Liver Cirrhosis: A Randomized Double-Blind Controlled Trial
Dietary supplement	HMB	University of Roma La Sapienza	NA	Sarcopenia, liver cirrhosis	Recruiting	NCT03892070	2019	2020	β-Hydroxy-β-methylbutyrate Supplementation and Physical Activity in Liver Cirrhosis: A Controlled Trial
Dietary supplement	Ensure Plus Advance, Ensure High Protein	Instituto Aragones de Ciencias de la Salud/Refbio2: Trans-Pyrenean Cooperation Network for Biomedical Research	NA	Sarcopenia, liver cirrhosis	Active, not recruiting	NCT03285217	2017	2019	HMB for Denutrition in Patients with Cirrhosis (HEPATIC)
Dietary supplement	Fresubin energy (dietary protein energy supplement)	Medical University of Graz	NA	Sarcopenia, liver cirrhosis	Recruiting	NCT03080129	2017	Ongoing	Microbiome and Sarcopenia in Patients with Liver Cirrhosis: A Prospective Controlled Cohort Study
Dietary supplement	Medically tailored meals, protein supplements	University of Michigan	NA	Sarcopenia, liver cirrhosis, hepatic encephalopathy, ascites	Recruiting	NCT04675775	2021	Ongoing	Medically Tailored Meals to Prevent Recurrent Hepatic Encephalopathy: The BRAINFOOD Pilot Trial
Multifactorial intervention	Home exercise, BCAA supplements, multispecies probiotic	Fundació Institut de Recerca de l’Hospital de la Santa Creu i Sant Pau	NA	Sarcopenia, liver cirrhosis, frailty syndrome (FS)	Recruiting	NCT04243148	2020	Ongoing	Frailty in Patients with Cirrhosis: Prognostic Value of the Phase Angle in Hospitalized Patients and Effect of a Multifactorial Intervention (Home Exercise, Branched-chain Amino Acids, and Probiotics)
Multifactorial intervention	Physical training program, behavioral modification therapy, nutritional consultation	University of Arkansas	NA	End-stage liver disease, liver transplant, sarcopenia, cirrhosis	Completed	NCT02776553	2016	2020	A Physical Activity Program in End-Stage Liver Disease: Pilot Study Assessing Changes in Physical Fitness, Sarcopenia, and the Metabolic Profile

Data were presented in the Clinicaltrials.gov on 1 February 2021. Abbreviation: BCAA, branched chain amino acid; HMB, β-hydroxy-β-methylbutyrate; NA, not applicable.

## Data Availability

Not applicable.

## Supplementary Materials

The following are available online at https://www.mdpi.com/2075-1729/11/3/250/s1, Table S1: Current and emerging treatment options for sarcopenia that have been observed in clinical trials.
