# Supplementary material for: Emerging Treatment Options for Sarcopenia in Chronic Liver Disease
Source: Life (Basel). 2021 Mar 17;11(3):250. doi: 10.3390/life11030250 (PMC8002763; doi:10.3390/life11030250)
Supplement: Supplementary file 1 [file life-11-00250-s001.pdf]

Table S1. Current and emerging treatment options for sarcopenia observed in clinical trials.

Target or Mechanism of action	Drug	Sponsor/Collaborator	Clinical Phase	Indication	Status	NCT number	Year		Title	Reference
							Start	End		
Activin receptor inhibitor	Bimagrumab (BYM338)	Novartis Pharmaceuticals	2	Sarcopenia	Completed	NCT01601600	2012	2013	A Randomized, Double-blind, Placebo-controlled Multi-center Study to Assess the Effects of BYM338 on Skeletal Muscle in Sarcopenic Adults With Mobility Limitations	[1]
Activin receptor inhibitor	Bimagrumab (BYM338)	Novartis Pharmaceuticals	2	Sarcopenia	Completed	NCT02333331	2014	2018	A 28 Week, Randomized, Double-blind, Placebo-controlled, Two-part, Multi-center, Parallel Group Dose Range Finding Study to Assess the Effect of Monthly Doses of Bimagrumab 70, 210, and 700 mg on Skeletal Muscle Strength and Function in Older Adults With Sarcopenia (InvestiGAIT)	[2]
Activin receptor inhibitor	Bimagrumab (BYM338)	Novartis Pharmaceuticals	2	Muscle Wasting (Atrophy) After Hip Fracture Surgery	Completed	NCT02152761	2014	2018	A 24-week Double-blind Treatment and 24-week Follow-up, Randomized, Multicenter, Placebo-controlled, Phase IIa/IIb Study to Evaluate Safety and Efficacy of i.v. Bimagrumab on Total Lean Body Mass and Physical Performance in Patients After Surgical Treatment of Hip Fracture	NA
Activin receptor inhibitor	Bimagrumab (BYM338)	Novartis Pharmaceuticals	2	Sarcopenia	Completed	NCT02468674	2015	2018	A 24 Week Off Drug Extension, Parallel Group, Study Assessing Durability of Effect on Skeletal Muscle Strength and Function Following a 6-month Double-blind, Placebo Controlled Study Evaluating Bimagrumab in Older Adults With Sarcopenia (InvestiGAIT Extension)	NA
Activin receptor inhibitor	Bimagrumab (BYM338)	Novartis Pharmaceuticals	2	Type 2 diabetes with obesity	Completed	NCT03005288	2017	2019	A Randomized, Subject- and Investigator-blinded, Placebo Controlled Study to Assess the Safety, Pharmacokinetics and Efficacy of Intravenous Bimagrumab in Overweight and Obese Patients With Type 2 Diabetes	[3]
Myostatin inhibitor	Trevogrumab (REGN1033)	Regeneron Pharmaceuticals/Sanofi	2	Sarcopenia	Completed	NCT01963598	2013	2015	A Randomized, Double-blind, Placebo-controlled, Multi-center Study of the Safety and Efficacy of 3-month Subcutaneous REGN1033 Treatment in Patients With Sarcopenia	NA
Myostatin inhibitor	Trevogrumab (REGN1033), REGN2477	Regeneron Pharmaceuticals	1	Healthy volunteers	Completed	NCT02943239	2016	2019	A Randomized, Double-Blind, Placebo-Controlled, Ascending Dose Study to Assess the Safety, Tolerability, and Pharmacodynamic Effects of REGN2477 Alone and in Combination With REGN1033 in Healthy Postmenopausal Women and Healthy Adult Men	NA
Myostatin inhibitor	Domagrozumab (PF-06252616)	Pfizer	2	Duchenne Muscular Dystrophy	Terminated	NCT02310763	2014	2018	A Phase 2 Study to Evaluate the Safety, Efficacy, Pharmacokinetics and Pharmacodynamics of PF-06252616 in Duchenne Muscular Dystrophy	[4]
Myostatin inhibitor	Domagrozumab (PF-06252616)	Pfizer	2	Duchenne Muscular Dystrophy	Terminated	NCT02907619	2016	2018	An Multicenter, Open-label Extension Study To Evaluate Safety Of PF-06252616 In Boys With Duchenne Muscular Dystrophy	[4]
Myostatin inhibitor	Domagrozumab (PF-06252616)	Kathryn Wagner/Pfizer	1b/2	Limb Girdle Muscular Dystrophy 2I (LGMD2I)	Completed	NCT02841267	2016	2019	A Phase 1b/2, Open-Label, Multiple Ascending Dose Study to Evaluate the Safety, Tolerability, Efficacy, Pharmacokinetics and Pharmacodynamics of PF-06252616 in Ambulatory Participants With LGMD2I	[5]
Myostatin inhibitor	ACE-083	Acceleron Pharma, Inc.	2	Facioscapulohumeral Muscular Dystrophy (FSHD)	Terminated	NCT02927080	2016	2019	A Phase 2 Randomized, Double-Blind, Placebo-Controlled Study of ACE-083 in Patients With Facioscapulohumeral Muscular Dystrophy	NA
Myostatin inhibitor	ACE-083	Acceleron Pharma, Inc.	2	Charcot-Marie-Tooth (CMT)	Terminated	NCT03124459	2017	2020	A Phase 2 Randomized, Double-Blind, Placebo-Controlled Study of ACE-083 in Patients With Charcot-Marie-Tooth Disease Types 1 and X	NA
Myostatin inhibitor	ACE-2494	Acceleron Pharma, Inc.	1	Healthy Volunteers	Completed	NCT03478319	2018	2019	A Phase 1, Randomized, Double-Blind, Placebo-Controlled, Dose-Ranging Study to Evaluate the Safety, Tolerability, Pharmacokinetics, and Pharmacodynamic Effects of ACE-2494 in Healthy Postmenopausal Women	NA
Myostatin inhibitor	RO7239361	Hoffmann-La Roche	2	Duchenne Muscular Dystrophy	Completed	NCT03039686	2017	2020	A Randomized, Double Blind, Placebo-Controlled, Study to Assess the Efficacy, Safety, and Tolerability of RO7239361 in Ambulatory Boys With Duchenne Muscular Dystrophy	NA
Testosterone	Testosterone, Recombinant human growth hormone	NIA	2	Sarcopenia	Completed	NCT00183040	2002	2007	Hormonal Regulators of Muscle and Metabolism in Aging (HORMA)	[6]

Testosterone	Testosterone, Vitamin D	University of Zurich/Besins Healthcare, Dr. Wild & Co.	3	Hypogonadism, Pre-frail Seniors	Terminated	NCT02419105	2015	2020	Effects of Transdermal Testosterone and/or Monthly Vitamin D on Fall Risk in Pre-frail Hypogonadal Seniors (Double Blind, 2x2 Factorial, Randomized Placebo-Controlled Clinical Trial)	NA
Testosterone	Testosterone, Gonadotropin-releasing hormone agonist (Goserelin; sex hormone inhibitor)	University of Nottingham	3	Sarcopenia	Unknown	NCT03054168	2016	2019	The Regulation of Skeletal Muscle Protein Synthesis by Systemic Hormones and Its Influence on Ageing and Anabolic Resistance	NA
Testosterone	Testosterone, Exercise	Washington University School of Medicine	3	Hip fracture, Sarcopenia	Recruiting	NCT02938923	2017	Ongoing	Combining Testosterone Therapy and Exercise to Improve Function Post Hip Fracture	NA
Testosterone	Testosterone enanthate, finasteride (5-alpha reductase inhibitor)	VA Office of Research and Development/Endo Pharmaceuticals, Merck Sharp & Dohme Corp.	2	Sarcopenia	Completed	NCT00475501	2007	2014	5-Alpha Reductase and Anabolic Effects of Testosterone	[7]
Testosterone	Testosterone, Anastrozole (Aromatase inhibitor)	NIA	2	Sarcopenia	Completed	NCT00104572	2004	2015	The Effects of Aromatase Inhibition and Testosterone Replacement in Sex Steroids, Pituitary Hormones, Markers of Bone Turnover, Muscle Strength, and Cognition in Older Men	[8]
SARM	MK-0773	Merck Sharp & Dohme Corp.	2	Sarcopenia	Completed	NCT00529659	2007	2009	A Phase IIa Randomized, Placebo-Controlled Clinical Trial to Study the Efficacy and Safety of MK-0773 in Patients With Sarcopenia	[9]
SARM	Ligandrol (LGD-4033/VK5211)	Viking Therapeutics, Inc.	2	Hip Fractures	Completed	NCT02578095	2015	2018	A Phase II, Randomized, Double-Blind, Parallel Group, Placebo- Controlled, Multi-Center Study to Explore the Efficacy, Safety and Tolerability of VK5211 in Subjects With Acute Hip Fracture	NA
SARM	LY2452473	Dana-Farber Cancer Institute/National Institute of Nursing Research	2	Prostate Cancer	Completed	NCT02499497	2016	2020	A Selective Androgen Receptor Modulator for Symptom Management in Prostate Cancer	NA
SARM	Enobosarm (GTx-024)	GTx	2	Stress Urinary Incontinence	Completed	NCT03241342	2017	2018	Double-Blind, Placebo-Controlled, Parallel Design, Phase 2 Study to Assess Clinical Activity and Safety of Enobosarm (GTx-024) in Postmenopausal Women With Stress Urinary Incontinence	NA
SARM	Enobosarm (GTx-024)	City of Hope Medical Center/National Cancer Institute	2	Androgen Receptor Positive/Metastatic Triple-Negative Breast Carcinoma	Active, not recruiting	NCT02971761	2017	Ongoing	A Phase 2 Clinical Trial of the Combination of Pembrolizumab and Selective Androgen Receptor Modulator GTX-024 in Patients With Metastatic Androgen Receptor Positive Triple Negative Breast Cancer	[10]
SARM	GSK2881078	GlaxoSmithKline/Parexel	2	Cachexia	Completed	NCT03359473	2018	2019	A Randomized, Double-blind (Sponsor Unblind), Placebo-controlled, Multi-centred Phase IIa Study to Evaluate the Safety and Efficacy of 13 Weeks of Once Daily Oral Dosing of the Selective Androgen Receptor Modulator GSK2881078 in Older Men and Post Menopausal Women With COPD and Muscle Weakness, Participating in Home Exercise	NA
DHEA	DHEA	Washington University School of Medicine	3	Sarcopenia, osteopenia	Completed	NCT00205686	2001	2005	DHEA+Exercise-Effect on Sarcopenia and Osteopenia of Aging	[11]
Ghrelin	Ghrelin	University of Pennsylvania	2	Sarcopenia, Frailty Syndrome	Completed	NCT01898611	2013	2016	Ghrelin Plus Strength Training in Frail Elderly Study	NA
Ghrelin	Anamorelin Hydrochloride	Tufts University/NIAMS	1	Sarcopenia, Osteopenia	Recruiting	NCT04021706	2019	Ongoing	Effect of a Ghrelin Receptor Agonist on Muscle and Bone	NA
GH secretagogue	ibutamoren mesylate (MK-0677)	University of Virginia	2	Aging	Completed	NCT00474279	1998	2004	Effects of an Oral GH Secretagogue (MK-677) on Body Composition and Functional Ability of Older Adults	[12]
GH secretagogue	ibutamoren mesylate (MK-0677)	Merck Sharp & Dohme Corp.	2	Hip fracture	Terminated	NCT00128115	2005	2007	Treatment of Sarcopenia in Post-Hip Fracture Patients (0677-032)	[13]
Insulin	Insulin Regular, L-NMMA, Sodium Nitroprusside	The University of Texas Medical Branch, Galveston/NIA	1	Sarcopenia	Completed	NCT00690534	2005	2012	Insulin and Sarcopenia in the Elderly	NA

Insulin/IGF-1 signaling	Ursolic Acid	Pusan National University Hospital	2	Sarcopenia	Completed	NCT02401113	2014	2015	Effect of Ursolic Acid of Loquat Extract on Function of Muscle in Human	[14]
Mas receptor activator	BIO101 (Sarconeos)	Biophytis	2	Sarcopenia	Active, not recruiting	NCT03452488	2018	2021	Safety and Efficacy of BIO-101 Oral Administration to Patients Suffering From Age-related SARcopenia, Including Sarcopenic Obesity, Aged ≥65 Years and at Risk of Mobility Disability (SARA-INT)	NA
AMPK activation/mTOR inhibition	Metformin	VA Office of Research and Development/Oregon Health and Science University	2	Sarcopenia, Prediabetes	Completed	NCT01804049	2014	2018	Metformin and Muscle in Insulin-resistant Older Veterans	NA
AMPK activation/mTOR inhibition	Metformin	The University of Texas Health Science Center at San Antonio/NIA	2	Frailty	Recruiting	NCT02570672	2016	Ongoing	Metformin for Preventing Frailty in High-risk Older Adults	NA
AMPK activation/mTOR inhibition	Metformin	University of New Mexico	3	Prediabetes, Aging	Active, not recruiting	NCT03309007	2017	Ongoing	A Double-Blind, Placebo-Controlled Trial of Anti-Aging, Pro-Autophagy Effects of Metformin in Adults With Prediabetes	NA
AT1 receptor inhibitor	Losartan	Johns Hopkins University/NIH, NIA	2	Sarcopenia	Completed	NCT01989793	2013	2016	A Study of Muscle Strength Maintenance in Older Adults	[15,16]
AT1 receptor inhibitor	Losartan	University of Florida/NIA, Abbott	2	Inflammation	Completed	NCT02676466	2016	2018	The ENRGISE (ENabling Reduction of Low-Grade Inflammation in SEniors) Pilot Study	[17]
AT1 receptor inhibitor	Losartan, Perindopril, hydrochlorothiazide	University of Alabama at Birmingham/University of Colorado, Translational Research Institute for Metabolism and Diabetes, Advent Health	2	Hypertension, Aging, Sedentary lifestyle	Recruiting	NCT03295734	2018	Ongoing	ACES - ACE Inhibitors Combined With Exercise for Seniors With Hypertension	[18]
Fast skeletal muscle troponin activator	reldesemtiv (CK-2127107)	Astellas Pharma Global Development, Inc./Cytokinetics	1b	Mobility Limitation	Terminated	NCT03065959	2017	2018	A Phase 1b Study to Assess the Anti-fatigability Effect of CK-2127107 in Elderly Male and Female Subjects With Limitations in Mobility	NA
mTORC1 inhibitor, relaxing smooth muscle of blood vessels	Rapamycin, Sodium nitroprusside	The University of Texas Medical Branch, Galveston/NIAMS	1	Sarcopenia	Completed	NCT00891696	2009	2015	Nutritional and Contractile Regulation of Muscle Growth (Cycle 2)	NA
Myoblast differentiation	Cetylpyridinium Chloride	Seoul National University Hospital	1	Sarcopenia	Unknown	NCT02575235	2015	2016	Randomized, Double Blinded, Placebo-controlled Trial to Assess the Preventive Effects of Cetylpyridinium Chloride on Sarcopenia	NA
Oxytocin	Oxytocin nasal spray	Sara Espinoza/The University of Texas	2	Sarcopenic obesity	Completed	NCT03119610	2017	2019	The Physiologic Effects of Intranasal Oxytocin on Sarcopenic Obesity	NA
Vitamin D analogue	Alfacalcidol	Indonesia University	3	Muscle Weakness	Completed	NCT02327091	2012	2012	Phase 3 Study of Vitamin D Analog (Alfacalcidol) on Muscle Strength in Elderly Indonesian Women Who Have Low Handgrip Strength	NA

Data were presented in the Clinicaltrials.gov on 1 Feb 2021.
Abbreviations: National Institute on Aging, NIA; National Institutes of Health, NIH; National Institute of Arthritis and Musculoskeletal and Skin Diseases, NIAMS; Dehydroepiandrosterone, DHEA; Growth hormone, GH; Insulin-like Growth Factor 1, IGF-1, 5' adenosine monophosphate-activated protein kinase, AMPK; angiotensin II type 1, AT1; mammalian target of rapamycin, mTOR

References

1. Rooks, D.; Praestgaard, J.; Hariry, S.; Laurent, D.; Petricoul, O.; Perry, R.G.; Lach-Trifilieff, E.; Roubenoff, R. Treatment of Sarcopenia with Bimagrumab: Results from a Phase II, Randomized, Controlled, Proof-of-Concept Study. *J Am Geriatr Soc* **2017**, *65*, 1988-1995, doi:10.1111/jgs.14927.
2. Rooks, D.; Swan, T.; Goswami, B.; Filosa, L.A.; Bunte, O.; Panchaud, N.; Coleman, L.A.; Miller, R.R.; Garcia Garayoa, E.; Praestgaard, J., et al. Bimagrumab vs Optimized Standard of Care for Treatment of Sarcopenia in Community-Dwelling Older Adults: A Randomized Clinical Trial. *JAMA Netw Open* **2020**, *3*, e2020836, doi:10.1001/jamanetworkopen.2020.20836.
3. Heymsfield, S.B.; Coleman, L.A.; Miller, R.; Rooks, D.S.; Laurent, D.; Petricoul, O.; Praestgaard, J.; Swan, T.; Wade, T.; Perry, R.G., et al. Effect of Bimagrumab vs Placebo on Body Fat Mass Among Adults With Type 2 Diabetes and Obesity: A Phase 2 Randomized Clinical Trial. *JAMA Netw Open* **2021**, *4*, e2033457, doi:10.1001/jamanetworkopen.2020.33457.
4. Wagner, K.R.; Abdel-Hamid, H.Z.; Mah, J.K.; Campbell, C.; Guglieri, M.; Muntoni, F.; Takeshima, Y.; McDonald, C.M.; Kostera-Pruszczyk, A.; Karachunski, P., et al. Randomized phase 2 trial and open-label extension of domagrozumab in Duchenne muscular dystrophy. *Neuromuscul Disord* **2020**, *30*, 492-502, doi:10.1016/j.nmd.2020.05.002.
5. Leung, D.G.; Bocchieri, A.E.; Ahlawat, S.; Jacobs, M.A.; Parekh, V.S.; Braverman, V.; Summerton, K.; Mansour, J.; Bibat, G.; Morris, C., et al. Longitudinal functional and imaging outcome measures in FKRP limb-girdle muscular dystrophy. *BMC Neurol* **2020**, *20*, 196, doi:10.1186/s12883-020-01774-5.
6. Yarasheski, K.E.; Castaneda-Sceppa, C.; He, J.; Kawakubo, M.; Bhasin, S.; Binder, E.F.; Schroeder, E.T.; Roubenoff, R.; Azen, S.P.; Sattler, F.R. Whole-body and muscle protein metabolism are not affected by acute deviations from habitual protein intake in older men: the Hormonal Regulators of Muscle and Metabolism in Aging (HORMA) Study. *Am J Clin Nutr* **2011**, *94*, 172-181, doi:10.3945/ajcn.110.010959.
7. Borst, S.E.; Yarrow, J.F.; Conover, C.F.; Nseyo, U.; Meuleman, J.R.; Lipinska, J.A.; Braith, R.W.; Beck, D.T.; Martin, J.S.; Morrow, M., et al. Musculoskeletal and prostate effects of combined testosterone and finasteride administration in older hypogonadal men: a randomized, controlled trial. *Am J Physiol Endocrinol Metab* **2014**, *306*, E433-442, doi:10.1152/ajpendo.00592.2013.
8. Dias, J.P.; Melvin, D.; Shardell, M.; Ferrucci, L.; Chia, C.W.; Gharib, M.; Egan, J.M.; Basaria, S. Effects of Transdermal Testosterone Gel or an Aromatase Inhibitor on Prostate Volume in Older Men. *J Clin Endocrinol Metab* **2016**, *101*, 1865-1871, doi:10.1210/jc.2016-1111.
9. Papanicolaou, D.A.; Ather, S.N.; Zhu, H.; Zhou, Y.; Lutkiewicz, J.; Scott, B.B.; Chandler, J. A phase IIA randomized, placebo-controlled clinical trial to study the efficacy and safety of the selective androgen receptor modulator (SARM), MK-0773 in female participants with sarcopenia. *J Nutr Health Aging* **2013**, *17*, 533-543, doi:10.1007/s12603-013-0335-x.
10. Yuan, Y.; Lee, J.S.; Yost, S.E.; Frankel, P.H.; Ruel, C.; Egelston, C.A.; Guo, W.; Gillece, J.D.; Folkerts, M.; Reining, L., et al. A Phase II Clinical Trial of Pembrolizumab and Enobosarm in Patients with Androgen Receptor-Positive Metastatic Triple-Negative Breast Cancer. *Oncologist* **2020**, 10.1002/onco.13583, doi:10.1002/onco.13583.
11. Villareal, D.T.; Holloszy, J.O. Effect of DHEA on abdominal fat and insulin action in elderly women and men: a randomized controlled trial. *JAMA* **2004**, *292*, 2243-2248, doi:10.1001/jama.292.18.2243.
12. Chapman, I.M.; Bach, M.A.; Van Cauter, E.; Farmer, M.; Krupa, D.; Taylor, A.M.; Schilling, L.M.; Cole, K.Y.; Skiles, E.H.; Pezzoli, S.S., et al. Stimulation of the growth hormone (GH)-insulin-like growth factor I axis by daily oral administration of a GH secretogogue (MK-677) in healthy elderly subjects. *J Clin Endocrinol Metab* **1996**, *81*, 4249-4257, doi:10.1210/jcem.81.12.8954023.
13. Adunsky, A.; Chandler, J.; Heyden, N.; Lutkiewicz, J.; Scott, B.B.; Berd, Y.; Liu, N.; Papanicolaou, D.A. MK-0677 (ibutamoren mesylate) for the treatment of patients recovering from hip fracture: a multicenter, randomized, placebo-controlled phase IIb study. *Arch Gerontol Geriatr* **2011**, *53*, 183-189, doi:10.1016/j.archger.2010.10.004.
14. Cho, Y.H.; Lee, S.Y.; Kim, C.M.; Kim, N.D.; Choe, S.; Lee, C.H.; Shin, J.H. Effect of Loquat Leaf Extract on Muscle Strength, Muscle Mass, and Muscle Function in Healthy Adults: A Randomized, Double-Blinded, and Placebo-Controlled Trial. *Evid Based Complement Alternat Med* **2016**, *2016*, 4301621, doi:10.1155/2016/4301621.
15. Burks, T.N.; Andres-Mateos, E.; Marx, R.; Mejias, R.; Van Erp, C.; Simmers, J.L.; Walston, J.D.; Ward, C.W.; Cohn, R.D. Losartan restores skeletal muscle remodeling and protects against disuse atrophy in sarcopenia. *Sci Transl Med* **2011**, *3*, 82ra37, doi:10.1126/scitranslmed.3002227.
16. Abadir, P.M.; Foster, D.B.; Crow, M.; Cooke, C.A.; Rucker, J.J.; Jain, A.; Smith, B.J.; Burks, T.N.; Cohn, R.D.; Fedarko, N.S., et al. Identification and characterization of a functional mitochondrial angiotensin system. *Proc Natl Acad Sci U S A* **2011**, *108*, 14849-14854, doi:10.1073/pnas.1101507108.

17. Pahor, M.; Anton, S.D.; Beavers, D.P.; Cauley, J.A.; Fielding, R.A.; Kritchevsky, S.B.; Leeuwenburgh, C.; Lewis, K.H.; Liu, C.K.; Lovato, L.C., et al. Effect of Losartan and Fish Oil on Plasma IL-6 and Mobility in Older Persons. The ENRGISE Pilot Randomized Clinical Trial. *J Gerontol A Biol Sci Med Sci* **2019**, *74*, 1612-1619, doi:10.1093/gerona/gly277.
18. Harper, S.A.; Baptista, L.C.; Roberts, L.M.; Wherry, S.J.; Boxer, R.S.; Hildreth, K.L.; Seay, R.S.; Allman, P.H.; Carter, C.S.; Aban, I., et al. Angiotensin Converting Enzyme Inhibitors Combined with Exercise for Hypertensive Seniors (The ACES Trial): Study Protocol of a Randomized Controlled Trial. *Front Med (Lausanne)* **2019**, *6*, 327, doi:10.3389/fmed.2019.00327.
